# The Role of IGL-2 Preservation Solution on Rat Livers during SCS and HOPE

**DOI:** 10.3390/ijms232012615

**Published:** 2022-10-20

**Authors:** Njikem Asong-Fontem, Arnau Panisello-Rosello, Mylène Sebagh, Mathilde Gonin, Joan Rosello-Catafau, René Adam

**Affiliations:** 1 Unité Chronothérapie, Cancers et Transplantation, Université Paris-Saclay, 94800 Villejuif, France; 2 Experimental Hepatic Ischemia-Reperfusion Unit, Institut d’Investigacions Biomèdiques de Barcelona (IIBB), Spanish National Research Council (CSIC), 08036 Barcelona, Catalonia, Spain; 3 Centre Hépato-Biliaire, APHP Hôpital Universitaire Paul Brousse, Université Paris-Saclay, 94800 Villejuif, France

**Keywords:** ischemia-reperfusion, glycocalyx, machine perfusion, fatty liver

## Abstract

The scarcity of livers for transplantation is rising, and new strategies to extend the donor pool are being explored. One solution is to use marginal grafts from extended criteria donors, presenting, for example, liver steatosis. As current preservation solutions (UW, HTK, and IGL-1) were mainly designed for static cold storage (SCS) only, IGL-2, a modified version of IGL-1, was developed to be suitable for SCS and dynamic preservation, such as hypothermic oxygenated perfusion (HOPE). In this study, we investigated the combined effect of IGL-2, SCS, and HOPE and compared it to the most used preservation solution (UW and Belzer MPS). Four experimental groups with six rats each were designed using Zucker rats. All groups underwent 24 h of SCS (in IGL-2 or UW) + 2 h of normothermic machine perfusion (NMP) at 37 °C to mimic transplantation. HOPE (IGL-2 or Belzer MPS) was performed before NMP on half of the rats. The IGL-2 group demonstrated lower transaminases and a significantly low level of glycocalyx proteins, CASP3, and HMGB1 in the perfusates. These data suggest the protective role of IGL-2 for fatty livers in preserving the endothelial glycocalyx, apoptosis, and inflammation.

## 1. Introduction

Surgeons and researchers have explored new strategies to expand the pool of donors and shorten the patient waitlist for liver transplantation [1]. One of the most promising approaches is the use of marginal grafts from steatotic livers or extended criteria donors (ECD) grafts from old donors (over age 60), cardiac death donors, or donors with early non-alcoholic fatty liver disease (NAFLD), also known as liver steatosis [2,3]. They all show a high vulnerability against ischemia-reperfusion injury (IRI), which describes the accumulation of lesions from the liver procurement in the donor (organ recovery, washout, and cold storage preservation) to liver transplantation in the recipient [4,5]. The graft’s static cold storage (SCS) in a cold (5 °C ± 3 °C) preservation solution is the most used strategy in clinical liver transplantation which is characterized by the deprivation of oxygen [6,7]. In this context, the composition of the preservation solution is crucial to prevent ischemia-reperfusion damage associated with liver transplantation, especially when steatosis is present.

According to the European Liver Transplant Registry (ELTR), the most used preservation solutions for liver grafts are UW, HTK, and IGL-1 [8]. The use of HTK has been limited, due to concerns emitted by the United Network for Organ Sharing (UNOS). The UW solution is currently the gold-standard preservation solution for livers [9,10]. Initially developed for pancreas transplantation in the late 1980s, it is characterized by its intracellular composition (high K^+^ and low Na^+^) and the presence of hydroxyethyl starch (HES), an oncotic agent. Even though the efficacy of HES in the reduction of edema has been widely demonstrated in multiple organs [11,12,13], it has also been involved in the hyperaggregation of red blood cells [14]. This last characteristic is highly harmful to graft washout and perfusion, especially in the context of steatotic livers where microcirculation is altered.

The IGL-1 solution is acknowledged to be an alternative to the UW solution. It has an extracellular composition (low K^+^/high Na^+^) and polyethylene glycol 35kDa (PEG35) as an oncotic agent. PEGs are neutral, water-soluble, non-toxic, and non-immunogenic polymers. The use of PEG was first tested by Robinson, who demonstrated its relevance in edema prevention [15]. Its efficacy in promoting hepatocyte cytoprotective factors was further confirmed in animal models and in humans [16,17]. PEG35 also increases NO production, a key player in vasodilation [18]. NO production improves microcirculation within the graft, therefore decreasing reperfusion injury [19,20]. For this reason, IGL-1 is positioned as an excellent alternative to UW as it overcomes one of its main limitations.

More recently, the appearance of promising dynamic perfusion strategies using normothermia and hypothermia conditions such as normothermic machine perfusion (NMP) and hypothermic oxygenated perfusion (HOPE) have been proposed as promising and valuable preservation strategies for increasing the preservation of the liver graft before transplantation. Both preservation strategies are based on the oxygenation of the liver graft using either a blood-based solution (NMP) or a transient oxygenation during hypothermic perfusion (HOPE).

In NMP, the liver is preserved at 37 °C and perfused with a blood-based solution. This technique allows the clinicians to perform real-time viability assessments of the liver and to incorporate additives, such as defatting agents, into the perfusate to reduce steatosis [21]. Even though this technique is key to assessing liver function and treating some metabolic dysfunction, it leads to a dysfunctional mitochondrial electron transport chain, causing reactive oxygen species (ROS) accumulation and activating inflammation pathways [22].

By contrast, the liver is perfused at 5 °C ± 3 °C under a continuous oxygen flow during HOPE. Hypothermia and oxygenation are crucial factors, allowing the metabolism to slow down and reducing the generation of ROS through the maintenance of the electron transport chain at basal levels [23,24]. Most of these preservation solutions include components that are aimed to focus on the same issues that are faced when dealing with the main harmful effects of SCS: hypothermic-induced cell swelling (e.g., oncotic agents, such as HES or PEG-35), minimizing intracellular acidosis (e.g., pH buffers), preventing injury from oxygen free radicals (e.g., antioxidants, such as glutathione or allopurinol) and providing substrates to regenerate ATP high-energy phosphate compounds (e.g., amino acids, ATP precursors, adenosine, and phosphate) during reperfusion.

However, in both HOPE and NMP, fluid dynamics play a role, leading to harmful effects on the endothelial glycocalyx matrix, the sugar layer covering the surface of the endothelial cells [25,26]. It mainly comprises proteoglycans (e.g., syndecan-1/SDC1) and glycoproteins (e.g., heparan sulfate/HPSG). The glycocalyx preservation is associated with endothelial cell integrity, the first cells affected by the liver dynamic preservation process. Previous studies have shown that the glycocalyx density is correlated to the shear stress caused by dynamic fluid perfusion, including organ washouts [27]. Moreover, glycocalyx disruption can play a role in the induction of inflammation and could contribute to the increase of IRI [28]. It has been demonstrated that increased SDC1 and HPSG were observed in the plasma of patients with end-stage liver diseases [29]. Recently, Schiefer et al. have proposed that the glycocalyx is a suitable marker in clinical liver transplantation [30]. Since PERF-GEN (Belzer MPS), a HES-based preservation solution, is the only dynamic perfusion solution available, a PEG-based solution can potentially improve dynamic perfusion outcomes. Moreover, adding PEG35 makes IGL-2 less viscous than PERF-GEN, which can be beneficial in protecting the glycocalyx.

The endothelial glycocalyx is also very sensitive to the shear stress alterations, which exert deleterious effects on the endothelial glycocalyx that are associated with alterations in the transduction mechanism changes, which will provoke concomitant glycocalyx degradation during HOPE in contrast to static preservation [31]. In this context, the density/viscosity of the perfusate will also be an influencing factor. With this in mind, HOPE is always thought to be combined with SCS because of the complex route from organ procurement to transplantation.

The present research investigates the efficiency of the IGL-2 solution, a modified version of IGL-1, where vasodilatation and antioxidant properties were boosted by increasing five-fold and three-fold the concentration of PEG35 and glutathione, respectively (Table 1). We hypothesized that these boosted properties are the most suitable ones for combining static preservation and dynamic perfusion ([32], p. 2, [33]). Moreover, the use of a unique solution for both the static and dynamic preservation solutions could greatly facilitate the logistical hurdles and avoid “interactions” that could be caused by the mixing of the different solutions when static cold storage is combined with HOPE using complete or partial liver grafts, as recently demonstrated by Mabrut et al. [34].

**Scheme 1:**
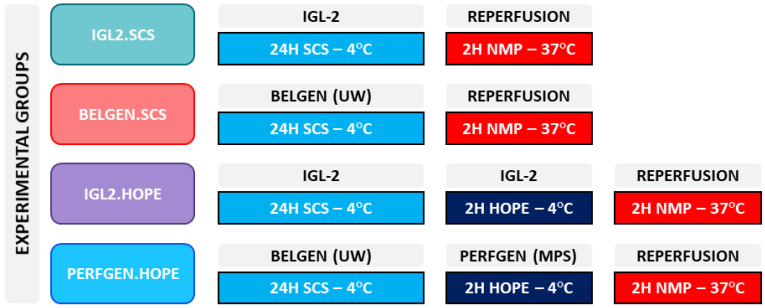
Experimental groups and study design description. Preservation solutions are highlighted in grey and black: IGL-2: Institut Georges Lopez-2; Reperfusion: albumin-based reperfusion solution; UW: University of Wisconsin; MPS: machine perfusion solution (Belzer MPS). Preservation protocols are highlighted in blue, dark blue, and red: SCS: static cold storage; NMP: normothermic machine perfusion; HOPE: hypothermic oxygenated machine perfusion.

In this study, we assessed the potential of IGL-2 as a unique solution for both static and dynamic preservation protocols (Scheme 1). Using the Zucker rat lineage, a rat model of the steatotic liver, we compared the IGL-2 solution to the BEL-GEN (UW composition) and PERF-GEN (Belzer MPS composition). After 24 h of static cold storage (for IGL2.SCS vs. BELGEN.SCS comparison) added to 2 h of HOPE (for IGL2.HOPE vs. PERFGEN.HOPE comparison) and 2 h of NMP (for all groups), we measured transaminases, lactates, and key markers of glycocalyx and apoptosis. For the first time, we compared IGL-2 to the Belzer MPS solution for fatty liver HOPE strategies. In this study, we demonstrated the suitability of using IGL-2 as an alternative to UW and Belzer MPS to improve the quality of steatotic graft preservation subjected to HOPE. Additionally, IGL-2 use permits the combination of hypothermic static and dynamic preservation strategies, thus simplifying the complicated logistics involved in clinical liver transplantation to better preserve the liver graft quality.

## 2. Results

### 2.1. Static Cold Storage

#### 2.1.1. Histology

**Figure 1:**
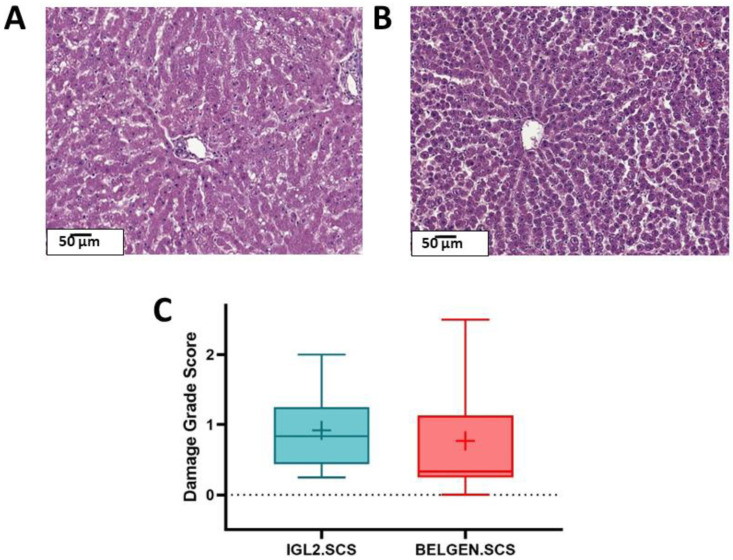
Liver histology after 24 h SCS + 2 h NMP. (**A**) IGL2.SCS (**B**) BELGEN.SCS (**C**) The damage grade score (DGS) was assessed by an expert pathologist and tended to be higher in the IGL2.SCS Group (*p* = 0.68) (Kruskal–Wallis test).

After two hours of reperfusion, liver biopsies were fixed in PFA for histological analysis (Figure 1). From a macroscopic standpoint, we observed the conservation of the hepatocellular architecture; more precisely, the hepatic lobules in both groups. To further characterize the livers’ preservation states, we calculated the damage grade score (DGS), a score based on inflammation, steatosis, perinuclear halo, and sinusoidal dilatation assessed by an expert pathologist. The livers preserved with BELGEN had a lower damage grade score compared to those preserved with IGL2. Highly variable levels of microsteatosis were observed among the tissues (0–20%) even though hepatectomy was performed in rats aged 9–10 weeks.

#### 2.1.2. Liver Function (Transaminases, Lactates)

**Figure 2:**
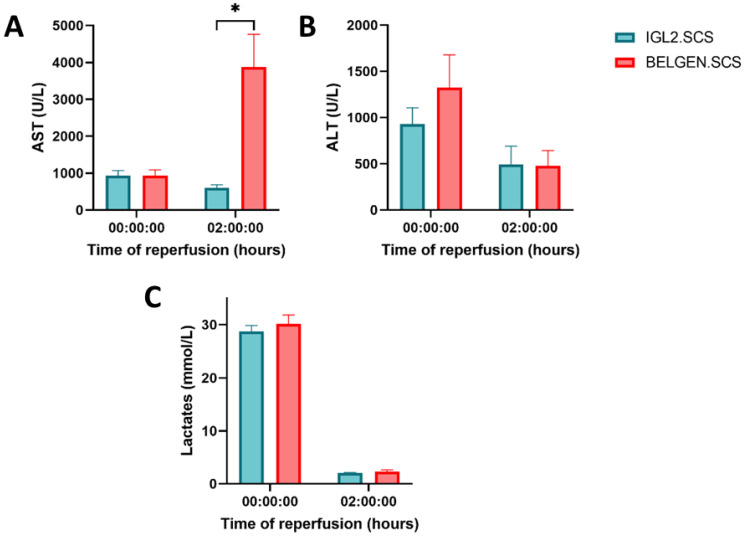
Hepatocellular injury markers quantification and anaerobic state evaluation. (**A**) Aspartate aminotransferase (AST) was significantly lower in the IGL2 group than in the BELGEN group after 2 h of reperfusion (*p* < 0.01). (**B**) Alanine aminotransferase (ALT) expression tended to be decreased in the IGL2 group compared to the BELGEN group after 2 h of reperfusion (*p* = 0.95). (**C**) No differences were observed in the lactate levels between the two groups. n = 6/group. * = *p* < 0.05.

Liver function was evaluated during two hours of reperfusion at 37 °C, following 24 h of SCS and 2 h of HOPE (Figure 2). The hepatocellular injury was assessed by quantifying AST and ALT transaminases as a function of time. The lactate levels indicated the anaerobic state of the hepatocytes over time. Increased levels of AST and ALT are correlated with liver injury. At T0, we observed no difference in AST levels. However, after 2 h of reperfusion, AST levels were significantly lower in IGL2.SCS (*p* < 0.01). ALT level, the liver-specific transaminase, decreased over time in both experimental groups. However, the difference observed is not significant. A significant decrease in lactates level was observed between T0 and T120. This is explained by the Ringer lactate rinse performed after the SCS phase. At T120, lactate levels decreased to reach the physiological range. No difference was observed in the lactate levels between the two groups.

#### 2.1.3. Glycocalyx

**Figure 3:**
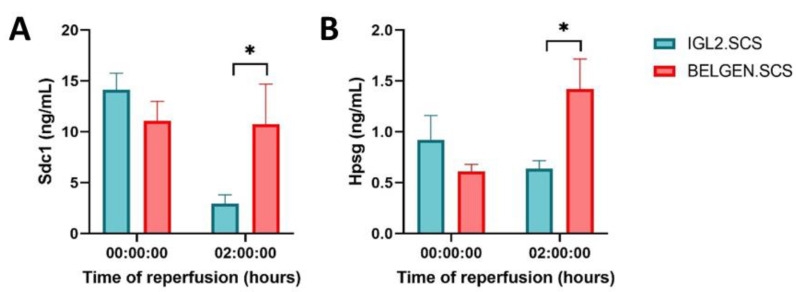
Glycocalyx integrity during reperfusion at 37 °C following 24 h of SCS. Proteins expression of SDC1 and HPSG by ELISA were measured from perfusates. (**A**) SDC1 levels were significantly lower after 2 h of reperfusion in the IGL2 group (*p* = 0.04). (**B**) HPSG levels were significantly lower after 2 h of reperfusion in the IGL2 group (*p* = 0.01). n = 6/group. * = *p* <0.05.

We assessed the evolution of two essential proteins (syndecan-1/SDC1 and heparan sulfate proteoglycan/HPSG) involved in glycocalyx, a thin glycoproteins layer at the luminal surface of the endothelium, which is protects the blood vessels. Their presence in the perfusate indicates a loss of glycocalyx integrity. The levels of SDC1 and HPSG were measured(Figure 3). At T0, we observe that SDC1 and HSPG tended to be increased in the IGL2 group. However, after 2 h of reperfusion, the trend was inverted. Indeed, SDC1 and HPSG were significantly lower in the IGL2 group, suggesting a preservation of the glycocalyx on the blood vessels.

#### 2.1.4. Apoptosis and Inflammation

**Figure 4:**
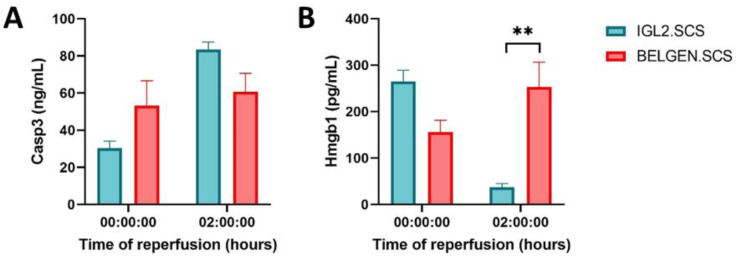
Expression of apoptosis-related marker active caspase 3 and inflammation marker HMGB1 in the perfusate during reperfusion following 24 h of SCS and 2 h of HOPE. (**A**) We observe no significant difference in the caspase 3 levels at T0 and T120. (**B**) HMGB1 (*p* < 0.01) is significantly less expressed in the IGL2 group after 2 h of reperfusion. n = 6/group. ** = *p* < 0.01.

Caspases are proteases involved in the regulation of apoptosis. The expression of caspase 3, an executioner caspase that is activated when cleaved, is used to assess and compare the levels of apoptosis in the two groups. Between 0 and 2 h, we observed an increase in caspase 3 levels in both groups (Figure 4). However, no significant differences were observed between the two experimental groups. High Mobility Group Box-1 (HMGB1) is a ubiquitous DNA-bound protein that acts as a cytokine when released by the nucleus in response to stress through the initiation of inflammation. At T0, the HMGB1 level was higher in IGL2.SCS compared to BELGEN SCS. However, after 2 h of reperfusion, the HMGB1 level was significantly lower in the IGL2.SCS compared to BELGEN.SCS. We observed that the HMGB1 level was significantly lower in the IGL2.SCS group compared to the BELGEN.SCS group.

### 2.2. Hypothermic Oxygenated Perfusion

#### 2.2.1. Histology

**Figure 5:**
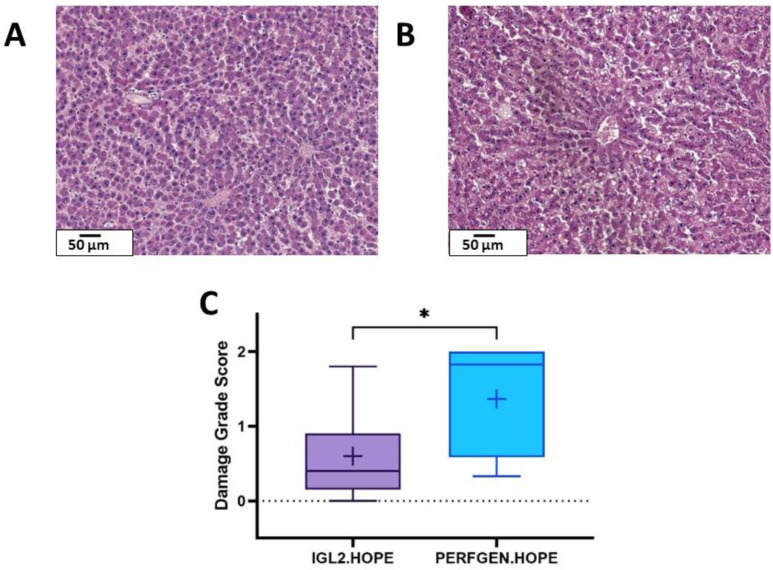
Liver histology after 24 h SCS + 2H HOPE + 2H NMP. (**A**) IGL2.HOPE (**B**) PERFGEN.HOPE (**C**) The damage grade score (DGS) was assessed by an expert pathologist and was significantly lower for the IGL2 group (*p* = 0.02). (Kruskal–Wallis test). * = *p* < 0.05.

The damage grade score (described in Figure 1) was also used to evaluate the preservation state of liver biopsies after hypothermic oxygenated perfusion. These groups demonstrated similar levels of steatosis. However, we observed more sinusoidal dilatation in the livers preserved with PERF-GEN compared to the IGL2.HOPE group (Figure 5). This partly explained the damage grade score differences, which were significantly lower in the IGL2.HOPE group compared to PERFGEN.HOPE (*p* = 0.02).

#### 2.2.2. Liver Function (Transaminases, Lactates)

**Figure 6:**
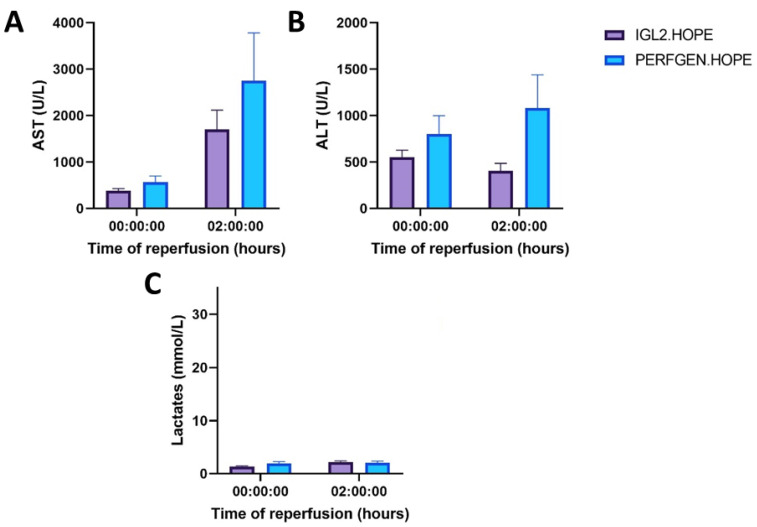
Hepatocellular injury markers quantification and anaerobic state evaluation. (**A**) Aspartate aminotransferase (AST) levels tended to be lower in the IGL2 group than in the PERFGEN group after 2 h of reperfusion (*p* = 0.53). (**B**) Alanine aminotransferase (ALT) expression tended to be decreased in the IGL2 group compared to the PERFGEN group after 2 h of reperfusion (*p* = 0.28). (**C**) No differences were observed in the lactate levels between the two groups. n = 6/group.

Transaminases and lactates were measured to assess hepatocyte injury biochemically. For both experimental groups, we observed that AST levels increased between 0 and 2 h (Figure 6). However, after two hours of reperfusion, AST levels seemed to be lower in the IGL2.HOPE groups. On the other side, we observed a slight decrease in alanine transferase (ALT), the liver-specific enzyme in the IGL2.HOPE group while in the PERFGEN. HOPE group ALT level increased over time. ALT decrease in IGL2.HOPE group could suggest a reduction in hepatocyte injury. Lactate levels, though staying constant in the physiological range over time, did not show any difference observed between the two groups.

#### 2.2.3. Glycocalyx

**Figure 7:**
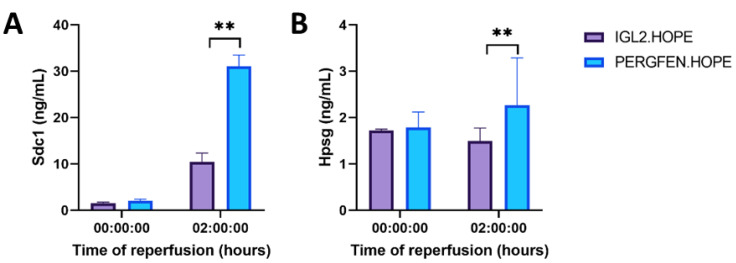
Glycocalyx integrity during reperfusion at 37 °C following 24 h of SCS and 2 h of HOPE. Proteins expression of SDC1 and HPSG by ELISA were measured from perfusates. (**A**) SDC1 levels were significantly lower after 2 h of reperfusion in the IGL2 group (*p* < 0.01). (**B**) HPSG levels were significantly lower after 2 h of reperfusion in the IGL2 group (*p* < 0.01). n = 6/group. ** = *p* < 0.01.

Glycocalyx preservation in the HOPE settings was assessed by measuring syndecan-1 and heparan sulfate levels in the perfusate at T0 and T120 during reperfusion. In both groups, the glycocalyx marker levels were similar at T0 (Figure 7). However, the SDC1 level drastically increased between T0 and T120 in both groups. On the other side, the HPSG level remained relatively constant between T0 and T120 for the IGL2.HOPE group while increasing for the PERFGEN.HOPE group. Overall, the level of SDC1 and HPSG was significantly lower in IGL2.HOPE compared to PERFGEN.HOPE after 2 h of reperfusion.

#### 2.2.4. Apoptosis and Inflammation

**Figure 8:**
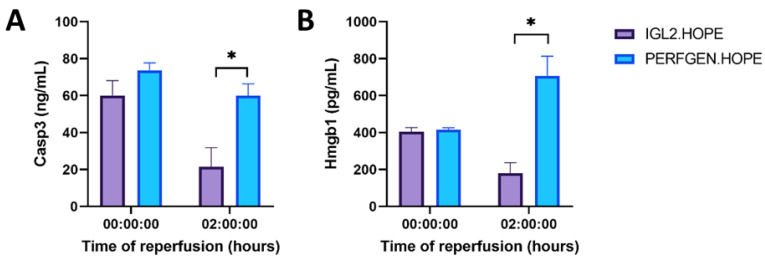
Expression of apoptosis-related marker active caspase 3 and inflammation marker HMGB1 in the perfusate during reperfusion following 24 h of SCS and 2 h of HOPE. Caspase 3 (*p* = 0.02) and HMGB1 (*p* = 0.03) were significantly less expressed in the IGL2 group after 2 h of reperfusion. n = 6/group. * = *p* < 0.05.

We measured programmed cell death using a cleaved caspase 3 marker. We observed that the caspase 3 level decreased over time in the IGL2.HOPE group compared to the PERFGEN.HOPE group where caspase 3 levels seemed to remain at a similar value (Figure 8). Moreover, we observed that caspase 3 was significantly higher in the PERFGEN.HOPE group after 2 h of reperfusion compared to the IGL2.HOPE group. Inflammation was measured using high mobility group box-1 (HMGB1). A reduction of the HMGB1 level between T0 and T120 was observed in the IGL2.HOPE group compared to PERFGEN.HOPE. Indeed, HMGB1 levels increased over time in the PERFGEN.HOPE group. Finally, the HMGB1 level was significantly lower in the IGL2.HOPE group compared to the PERFGEN.HOPE group after two hours of reperfusion.

### 2.3. Edema

**Figure 9:**
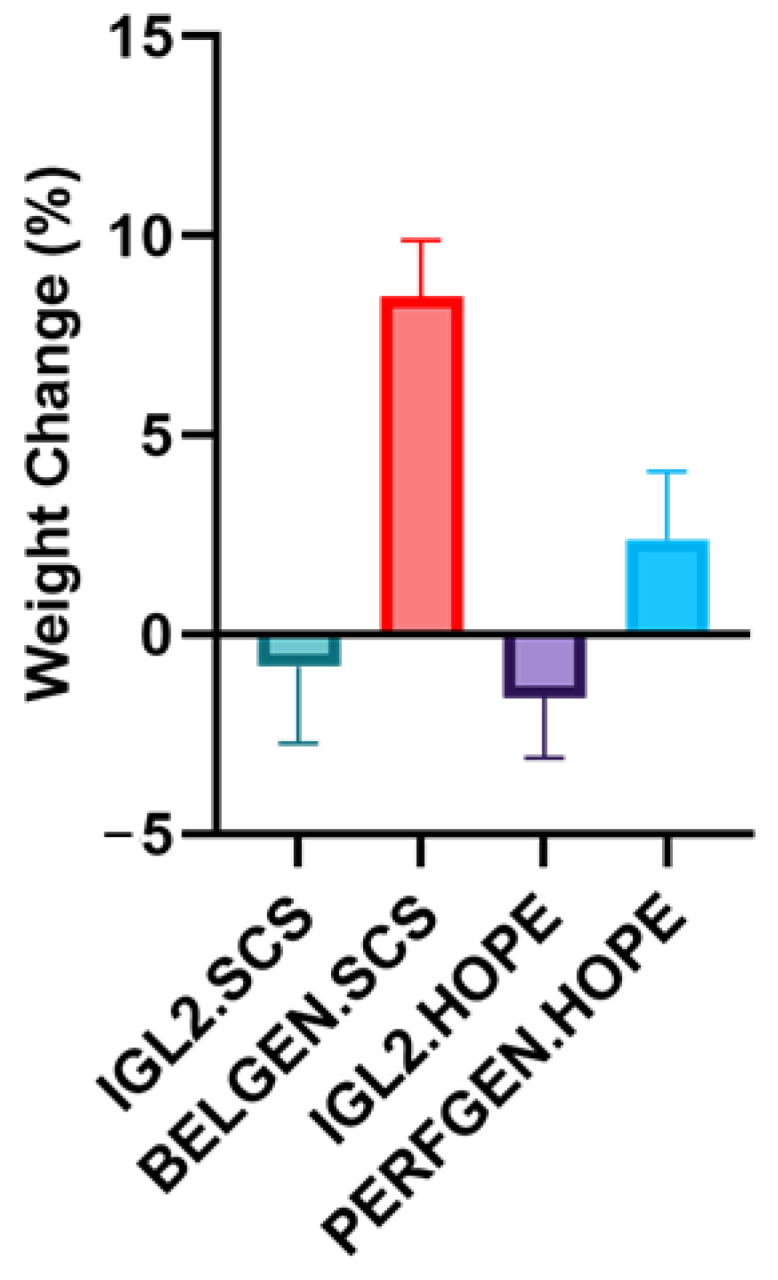
The percentage of weight change between the PRE-SCS and POST-SCS phases shows that the livers from the IGL2 groups seemed to have lost weight after NMP throughout preservation. In contrast, the livers from the BELGEN and PERFGEN groups tended to follow the opposite trend. n = 6/group.

Livers were weighed at three time points of preservation: before SCS, after SCS, and after reperfusion at 37 °C (POST-NMP) (Figure 9). We used weights as a probe of water retention and edema formation. We observed that IGL2 preserved livers’ weight change was negative, while BELGEN and PERFGEN livers’ weight change was positive between the PRE-SCS and POST-NMP phases. In addition, we observed that the BELGEN.SCS group demonstrated the highest weight gain throughout the preservation process. The IGL2.HOPE group seemed to have retained the lowest amount of water during preservation.

## 3. Discussion

Improving graft preservation during liver transplantation is key to reducing ischemia-reperfusion injuries. Even though healthy livers can better cope with preservation steps, sensitive organs, such as steatotic livers, need to be handled more carefully. New strategies to protect these fragile grafts need to be developed. To optimize liver preservation, two parameters come into play: the protocol used (whether it is static or dynamic) and the preservation solution used. Our present work aimed to assess the preservation of the endothelial glycocalyx during IRI in livers subjected to 24 h of cold ischemia and 24 h of cold ischemia + 2h of hypothermic oxygenated perfusion (HOPE) using three different preservation solutions: BEL-GEN (UW composition), PERF-GEN (Belzer MPS composition), and IGL-2.

Our study assessed transaminase (AST, ALT) levels after 2 h of reperfusion at 37 °C, mimicking the transplantation. In static or dynamic settings, we observed a lower level of transaminases in the IGL-2 groups compared to PERFGEN.HOPE and BELGEN.SCS, suggesting minimal protein leakage and hepatocyte membrane damage (Figure 2 and Figure 6). These results are consistent with the histological analysis showing a preserved structure of hepatic lobule architecture among all groups, though being more pronounced in the IGL2.HOPE group (Figure 1 and Figure 5). The previous observations can also be explained by the relatively mild level of steatosis within the rat lineage utilized for the analysis.

For this reason, the liver might not be categorized as a “steatotic liver” in humans. Indeed, it is well known that the HOPE protocol for a healthy liver with a low steatosis level does not significantly improve the preservation quality. Indeed, healthy livers perform the same way after static cold storage only and HOPE [35]. This is the reason why HOPE is indicated mainly for sensitive grafts. A study investigating IGL-2 efficacy in healthy rats has demonstrated similar transaminase levels [32].

The glycocalyx is a layer of proteoglycans (syndecan-1/SDC1, glypican-1/GCP1) and glycoproteins (heparan sulfate/HPSG) covering the luminal endothelial surface. It is a critical player in microvascular homeostasis and integrity [36]. Many stress or pathologic conditions, such as sepsis or ischemia-reperfusion injury, are a result of glycocalyx degradation [37]. During cold ischemia, the Kupffer cells (liver resident macrophages) are activated, and the endothelial cells release reactive oxygen species and inflammatory mediators [38]. The liberation of these two factors can damage the glycocalyx’s structure and integrity. A damaged glycocalyx facilitates the recruitment of immune cells, and the degradation of SDC1 and HPSG can cause vasoconstriction and tissue edema.

In our model, SDC1 and HPSG were measured during normothermic reperfusion. The release of glycocalyx molecules in the perfusate was significantly higher in the BEL-GEN and PERF-GEN groups after 2 h of reperfusion (Figure 3 and Figure 7). This suggests that there is improved preservation of the glycocalyx integrity in the IGL-2 groups. However, we observed that the glycocalyx markers level tended to be lower in the IGL2.SCS compared to the IGL2.HOPE. As the IGL2.HOPE group had 2 extra hours of perfusion due to the HOPE protocol; the glycocalyx was more exposed to shear stress. Therefore, seeing more SDC1 and HPSG in the IGL2.HOPE vs. the IGL2.SCS was an expected result.

We also observed that the SDC1, HPSG, and HMGB1 tended to be higher in the IGL2.SCS compared to BELGEN at T0. An inverted trend was observed at T120 for all three markers. The substantial increase of this marker after SCS could be due to the different pressure applied manually on the syringe during the Ringer lactate rinse right after SCS. Indeed, this manual rinse could be the origin of the different shear stresses in the vessels. During the reperfusion at 37 °C, all flows and pressure were maintained automatically at the same range. Therefore, it might be more sensitive to interpret the levels after 2 h of normothermic perfusion as the shear stress bias is removed.

Furthermore, an undamaged glycocalyx regulates vessel permeability and inhibits red blood cell coagulation. As a thin barrier between blood vessels and tissue, it controls inflammatory responses by regulating endothelial adhesion and migration of lymphocytes [39]. The lower viscosity of IGL-2 could also explain this compared to the Belzer MPS, which reduces the shear stress and preserves the luminal barrier (Table 1). Altogether, these results are in agreement with the results of Schiefer et al., which confirm that the quality of the glycocalyx is key to determining the capability of the graft to recover from IRI [30].

The present work also analyzed the active-caspase 3 and HMGB1 key markers of apoptosis induction and early inflammation, respectively. Both markers are known to be upregulated in the context of IRI. Indeed, several pharmacological markers have been linked to the decrease of liver IRI through the inhibition of apoptosis and the HMGB1 signaling pathway [40,41,42]. In our model, we observed a significant reduction of both markers in the IGL2.HOPE group compared to the PERFGEN.HOPE, which suggests that the previously described pathways were downregulated. At the same time, the HMGB1 was significantly lower in the IGL.SCS group vs. the BELGEN.SCS; we observed no difference for the apoptosis marker. It was expected that we would observe higher apoptosis levels in the static groups. Indeed, these groups were less exposed to oxygen than the HOPE groups. It is well known that anaerobic metabolism leads to higher apoptosis levels due to the increasing production of ROS [43]. We observed that HMGB1 values were lower overall in the SCS group than in the HOPE group. This correlates with the improved preservation of the glycocalyx in the SCS group. Indeed, previous studies demonstrated that the preservation of the glycocalyx integrity correlates with decreased inflammation [44,45].

Edema prevention is a key property of solid organ preservation; that is why most preservation solutions include an impermeant substance (Table 1) (raffinose, lactobionate) to counteract cell swelling [46]. Our study used liver weights to assess edema formation between the PRE-SCS and POST-NMP phases. We observed an increased weight loss in the IGL-2 groups compared to BEL-GEN and PERF-GEN groups (Figure 9). Edema is also controlled by the addition of oncotic agents in preservation solutions, which were PEG35 and HES, respectively, in IGL-2 and BEL-GEN/PERF-GEN. Our results suggest that PEG35 is an alternate oncotic agent, as it seems to decrease edema throughout the preservation steps. This result is consistent with previous studies, which described that the choice of colloids is key to providing long-term protection for graft injury and positioned PEG-based solutions as more efficient [47,48,49]. Several research groups have also demonstrated that PEG35 contributes to mitochondria preservation, endothelial glycocalyx preservation [50], transaminases reduction [32], NO generation (microcirculation improvement) [51], and could improve graft rinsing [52]. PEG components are also linked to the activation of anti-inflammatory pathways [53,54]. This statement is consistent with our results, where inflammation is significantly decreased in both IGL-2 preserved groups (PEG35-based solution).

Other studies exploring IGL-2 efficacy on healthy rats and fatty livers have demonstrated that the IGL-2 group demonstrated a lower level of glutamate dehydrogenase, a mitochondrial damage marker, vs. Belzer MPS [55]. Higher levels of aldehyde dehydrogenase 2 (ALDH2), a mitochondrial enzyme, were observed in the IGL-2 group vs. the Belzer MPS [56]. These results suggest that the IGL-2 solution confers better mitochondrial protection than the Belzer MPS, which is key to mitigating oxidative stress. To better characterize mitochondrial protection in further studies, FMN and NADH (components of the mitochondrial electron transport chain) can be measured. Indeed, previous studies have shown that FMN release during reperfusion is linked to mitochondrial ROS release and inflammation activation [24]. In this study, we have only measured markers during normothermic reperfusion; however, FMN and NADH levels during HOPE have the potential to predict liver function before implantation. Therefore, it could be of interest to quantify all the markers presented in this study during HOPE and better characterize their behavior in hypothermic conditions.

Finally, the selected ischemia-reperfusion injury markers used in this study, namely, the histological damage grade score, transaminases, glycocalyx, inflammation, and apoptosis markers, all seemed to position IGL-2 as the most suitable solution for HOPE performed on livers. Indeed, the most significant results favoring an optimized graft quality were observed for this experimental group, closely followed by IGL-2 in static cold storage settings. The only marker positioning IGL-2 as better in SCS conditions were the transaminases. Indeed, when comparing the transaminases of IGL-2, HOPE vs. IGL-2.SCS, we observed lower levels in the SCS group. This suggests increased liver injury in the IGL2.HOPE group, which is in contradiction with all the other IRI markers. This might question the relevance of the transaminase marker in assessing graft quality. Indeed, in physiological conditions, AST/ALT is key to analyzing liver function, but in an ex vivo setting, it might not be as sensitive.

To conclude, we have demonstrated that IGL-2 can be used for static and dynamic preservation, avoiding the mixture of preservation solutions currently happening when performing HOPE sequentially using UW and Belzer MPS. Having a unique solution for both protocols greatly facilitates the logistics and the time of liver preservation. All key markers of IRI analyzed in this study supported the use of IGL-2 vs. BELGEN in static settings and PERF-GEN in the HOPE setting. Indeed, not only does IGL-2 favor hepatocyte integrity and limit apoptosis and inflammation, but it also protects the glycocalyx barrier of marginal grafts. Further investigations will need to be conducted on other critical pathways involved in ischemia-reperfusion injury to investigate other functions in which IGL-2 is involved.

## 4. Materials and Methods

### 4.1. Animals

Homozygous male Zucker [Crl: ZUC(Orl)Leprfa] rats, aged 9–10 weeks, were purchased (Charles River, Lyon, France). The rats were housed in separate cages with a closed ventilation system, two rats per cage, in a room with controlled humidity and temperature and a 12 h light cycle. Water and food were provided ad libitum. The rats presented a rate of steatosis between 5% and 50%. Rats were chosen randomly.

### 4.2. Liver Procurement

Liver procurement was performed in a sterile room, under general anesthesia, through a silicon mask at 2 L/min airflow and isoflurane at 4% for induction and then at 2% for maintenance. The gastrointestinal tract was covered with wet gauze through a midline incision and placed to the left. The hepatic ligaments were cut on the left section of the diaphragm, and then the right suprarenal vein was closed with 8–0 non-absorbable polypropylene sutures. Heparin (0.3 UI/gr) was injected intravenously. The liver was rinsed through the abdominal aorta with 50 mL of preservation solution (PS) using a syringe pump (Pilote C^®^, Fresenius, France). Before procurement, the liver was flushed with 20 mL of PS through the portal vein at 5 °C ± 3 °C.

### 4.3. Experimental Groups

Four experimental groups (n = 6/group) were designed (Scheme 1) to compare the IGL-2 preservation solution to the gold standard of static preservation, BEL-GEN^®^ (Institut Georges Lopez, France), and the gold standard of dynamic preservation, PERF-GEN^®^ (Institut Georges Lopez, France). BEL-GEN and PER-GEN have the same composition as the original ones developed at the University of Wisconsin by F.O. Belzer and J. Southard, respectively, named UW and UW-MPS.

For static preservations groups, livers were preserved for 24 h in static cold storage (SCS) using IGL-2 (IGL2.SCS) or BEL-GEN (BELGEN.SCS) at 5 °C ± 3 °C. For dynamic preservation groups, livers underwent 2 h of hypothermic oxygenated perfusion (HOPE) at 5 °C ± 3 °C using IGL-2 (IGL2.HOPE) or PERFGEN (PERFGEN.HOPE) after 24 h of SCS at 5 °C ± 3 °C.

All livers were rinsed with 20 mL of Ringer lactate through the portal vein cannula to meet the clinical practice. To mimic liver transplantation, all livers underwent 2 h of normothermic oxygenated perfusion (NMP) using a perfusate composition previously described in Berendsen et al. [57]. The perfusate was collected during NMP at T0 and T120. Tissue samples were taken after the experiments. All samples were flash-frozen in liquid nitrogen and kept at −80 °C for further analysis.

### 4.4. Hypothermic Oxygenated Perfusion

**Figure 10:**
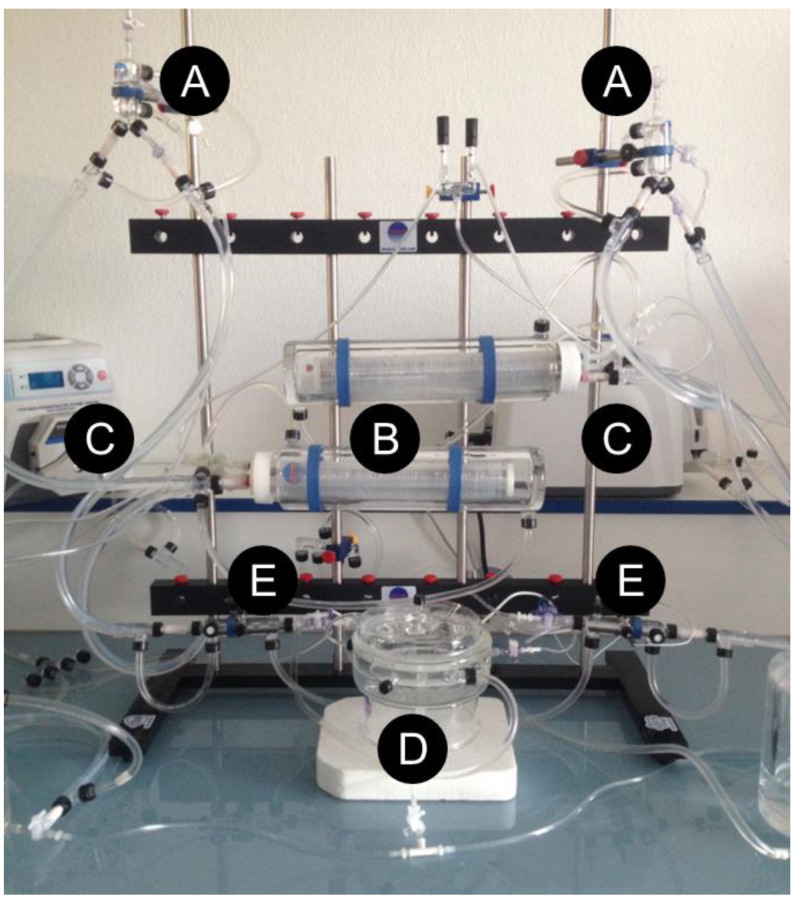
Experimental ex vivo machine perfusion system. Ex vivo machine perfusion system is adapted from liver/kidney systems (**A**) Bubble trap; (**B**) Membrane oxygenating; (**D**) Liver perfusion chamber (Radnoti LLC, Covina, CA, USA) assembled by the author (NA) (**C**) Peristaltic pump (MasterFlex^®^, Thermo Fisher Scientific, Waltham, MA, USA); (**E**) Flow sensor (TS410 Tubing Module, Transonic Systems Inc., Ithaca, NY, USA), a water bath circulator pump (9102A12E, 6-liter High-Stability Digital Controller Refrigerated/Heated Circulating Bath, Polyscience, Niles, IL, USA), and an oxygen bottle (95% O_2_/5% CO_2_, Air Liquide, Paris, France).

Hypothermic machine perfusion was performed according to previous studies using an in-house machine perfusion system [58,59]. We used the same machine perfusion system for HOPE and normothermic reperfusion, as shown below (Figure 10).

### 4.5. Liver Functional Test

Analysis of perfusate alanine aminotransferase (ALT)-aspartate aminotransferase (AST) was performed at the clinical laboratory at our institution using Siemens Healthcare Diagnostics-ADVIA^®^ Chemistry Systems AST and ALT reagents.

### 4.6. ELISA

Quantification of syndecan-1 (E-EL-R0996, Elabscience, Houston, TX, USA), heparan sulphate proteoglycan (E-EL-R0491, Elabscience, Houston, TX, USA), high mobility group protein B1 (E-EL-R0505, Elabscience, Houston, TX, USA), and active caspase 3 (AMS.E02A0015, AMSBIO, Oxfordshire, UK) in perfusates were obtained according to the manufacturer’s instructions. Optical density at 450 nm was quantified using a Modulus II Microplate Reader (Turner Biosystems, Sunnyvale, CA, USA).

### 4.7. Histology

Liver tissues were fixed in 10% paraformaldehyde and 3 μm sections were stained with haematoxylin-eosin saffron (HES) according to standard procedures. The damage grade score was based on necrosis and coagulation, acute necrosis, disorganization, granular precipitate, sinusoidal dilatation, eosinophilic deposits, number of cells remaining in vessels, and inflammation. An expert pathologist carefully measured each component and each was given a mark between 0–3 (0: absence; 1: low presence; 2: mild presence; and 3: high presence). Histologic slides were scanned using Lamina (Perkin Meyer, Waltham, MA) and analyzed with CaseViewer 1.4 software (3DHISTECH, Budapest, Hungary).

### 4.8. Statistics

Data from the damage grade scores were compared statistically using a nonparametric one-way ANOVA test (Kruskal–Wallis test) with Dunn’s post hoc analysis. All data are expressed as means (± SD or ± SEM). Data were compared statistically using multiple pairwise t-tests (normality tested with Shapiro–Wilk test). *p* < 0.05 was considered as significant. (GraphPad Prism version 8.0.0 for Windows, GraphPad Software, San Diego, CA, USA, https://www.graphpad.com, accessed on 31 August 2022).

## Figures and Tables

**Table 1:** Composition of IGL-2, BEL-GEN (UW), PERF-GEN (Belzer MPS).

	IGL-2	BEL-GEN (UW)	PERF-GEN (Belzer MPS)
**Electrolytes (mmol/L)**			
K^+^	25	125	25
Na^+^	125	30	120
Mg^2+^	5	5	5
SO_4_^2−^	5	5	5
Ca^+^	0.5	x	x
Zn^2+^	0.091	x	x
**Buffers (mmol/L)**			
Phosphate	25	25	25
HEPES	10	x	x
Histidine	30	x	x
**Impermeants (mmol/L)**			
Mannitol	60	X	30
Lactobionic acid	100	100	x
Dextrose	x	x	10
Ribose	x	x	5
Gluconate	x	x	85
**Colloids (g/L)**			
Hydroxyethyl starch (HES)	x	50	50
Polyethylene glycol 35 (PEG-35)	5	x	x
**Antioxidants**			
Glutathione (g/L)	9	3	3
**Metabolic precursors (mmol/L)**			
Adenosine	5	5	x
Adenine	x	x	5
NaNO_2_ (nmol/L)	50	x	x
pH	7.4	7.4	7.4
Osmolarity (mosmol/L)	360	320	320
Viscosity (Cp)	1.7	x	2.6

## Data Availability

The data presented in this study are available on request from the corresponding author.

## Abbreviations

ALT	Alanine Amino Transferase
AST	Aspartate Amino Transferase
BELZER MPS	Belzer Machine Perfusion Solution
DGS	Damage Grade Score
ECD	Extended Criteria Donors
ELTR	European Liver Transplant Registry
HES	Haematoxylin-Eosin Saffron (Coloration Agent)
HES	Hydroxy Ethyl Starch (Oncotic Agent)
HMGB1	High Mobility Group 1
HOPE	Hypothermic Oxygenation Perfusion
HSPG	Heparan Sulfate Proteoglycan
IGL	Institut Georges Lopez
IRI	Ischemia-Reperfusion Injury
NAFLD	Non-Alcoholic Fatty Liver Disease
NMP	Normothermic Machine Perfusion
NO	Nitric Oxide
PEG	Poly Ethylene Glycol
PFA	Paraformaldehyde
SCS	Static Cold Storage
SD	Standard Deviation
SDC1	Syndecan-1
UNOS	United Network of Organ Sharing
UW	University of Wisconsin
